# Iron stimulates plasma-activated medium-induced A549 cell injury

**DOI:** 10.1038/srep20928

**Published:** 2016-02-11

**Authors:** Tetsuo Adachi, Saho Nonomura, Minori Horiba, Tasuku Hirayama, Tetsuro Kamiya, Hideko Nagasawa, Hirokazu Hara

**Affiliations:** 1 Laboratory of Clinical Pharmaceutics, Gifu Pharmaceutical University, Gifu 501-1196, Japan; 2 Laboratory of Pharmaceutical and Medicinal Chemistry, Gifu Pharmaceutical University, Gifu 501-1196, Japan

## Abstract

Non-thermal atmospheric pressure plasma is applicable to living cells and has emerged as a novel technology for cancer therapy. Plasma has recently been shown to affect cells not only by direct irradiation, but also by indirect treatments with previously prepared plasma-activated medium (PAM). Iron is an indispensable element but is also potentially toxic because it generates the hydroxyl radical (•OH) in the presence of hydrogen peroxide (H_2_O_2_) via the Fenton reaction. The aim of the present study was to demonstrate the contribution of iron to PAM-induced A549 adenocarcinoma cell apoptosis. We detected the generation of •OH and elevation of intracellular ferrous ions in PAM-treated cells and found that they were inhibited by iron chelator. The elevations observed in ferrous ions may have been due to their release from the intracellular iron store, ferritin. Hydroxyl radical-induced DNA injury was followed by the activation of poly(ADP-ribose) polymerase-1, depletion of NAD^+^ and ATP, and elevations in intracellular Ca^2+^. The sensitivities of normal cells such as smooth muscle cells and keratinocytes to PAM were less than that of A549 cells. These results demonstrated that H_2_O_2_ in PAM and/or •OH generated in the presence of iron ions disturbed the mitochondrial-nuclear network in cancer cells.

Plasma is an ionized gas composed of positive/negative ions, electrons, radicals, uncharged (neutral) atoms and molecules, and UV photons^1^. Its radiation has been shown to generate some short- and long-lived molecules such as reactive oxygen and nitrogen species (RONS) mainly from oxygen and nitrogen in atmospheric air or solution^2^. Non-thermal atmospheric pressure plasma is applicable to living cells and tissues^1^ and has emerged as a novel technology for medical applications including cancer treatments^1^,^3^,^4^. Recent studies reported that plasma affected cancer cells not only directly, but also by the indirect treatment of cells with previously prepared medium irradiated by plasma, termed plasma-activated medium (PAM)^4^,^5^,^6^,^7^. The relatively short-lived RONS produced in medium by plasma irradiation may be converted to other relatively long-lived species such as hydrogen peroxide (H_2_O_2_), nitrate/nitrite (NO_x_), and other unknown species, which endow PAM with high and sustainable reactivity^5^,^8^,^9^. We recently reported that PAM functioned as a donor of reactive species, mainly H_2_O_2_, and induced apoptosis in the A549 human lung adenocarcinoma epithelial cell line and a few other cancer cell lines, and the addition of not only antioxidants, but also iron chelators to PAM significantly attenuated reductions in A549 cells viability^10^.

Iron is an indispensable element for living organisms. However, it is also potentially toxic because excess levels lead to the generation of the hydroxyl radical (•OH) in the presence of H_2_O_2_ via the Fenton reaction. •OH is the most harmful reactive oxygen species (ROS) that reacts at a diffusion-controlled rate with all biomolecules^11^. It has the ability to react with all components of DNA, damaging the purine and pyrimidine bases as well as the deoxyribose backbone^12^.

Ferritin is an iron storage protein that plays crucial roles in the homeostasis of cellular iron and protection of cells against the potential toxic effects of iron^13^,^14^. The antioxidant nature of ferritin has been demonstrated not only *in vitro*, but also *in vivo* in conditional ferritin knockout animals^15^. Ferritin is composed of 24 subunits of H and L chains, which assemble to form a protein shell, in which up to 4500 atoms of iron may be stored. A previous study reported that ferritin was degraded under some stress conditions, such as oxidative stress, infections, and iron deficiencies^14^.

The aims of the present study were to demonstrate the contribution of iron to the amplification of PAM’s inhibitory effects on A549 cell survival and also to elucidate the signaling mechanism responsible for cell death involving intracellular iron.

## Results

### Effects of iron ion chelators on PAM-induced cell injury

**Figure 1:**
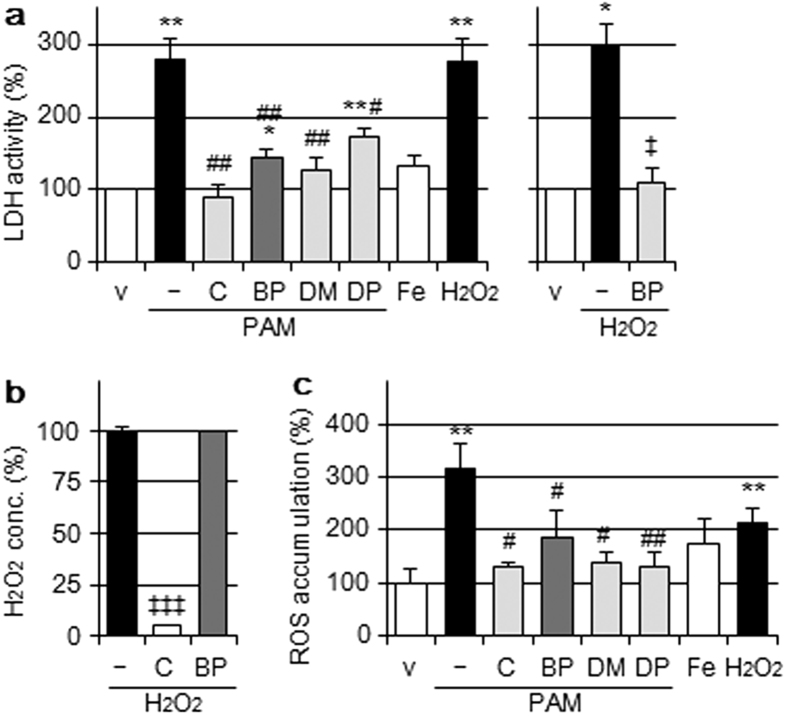
Effects of iron ion chelator and other reagents on PAM-induced cell injury. (**a**) Left: A549 cells were treated with DMEM (v); PAM in the presence or absence of catalase (C, 50 U/mL), BP (200 μM), DMTU (DM, 10 mM), or DPQ (DP, 20 μM); FeCl_2_-supplemented DMEM (Fe, 100 μM) or H_2_O_2_-supplemented DMEM (1 mM) for 6 h in a CO_2_ incubator, followed by the assay of LDH activity released into the conditioned medium. Right: A549 cells were treated with DMEM (v); H_2_O_2_-supplemented DMEM (1 mM) in the presence or absence of BP (200 μM) for 6 h, followed by the assay of LDH activity. Data are shown as means ± SD (n = 3). *p < 0.05, **p < 0.01 vs. DMEM only (v), ^#^*p* < 0.05, ^##^*p* < 0.01 vs. PAM only, ^‡^*p* < 0.05 vs. H_2_O_2_-supplemented DMEM only. (**b**) Catalase (50 U/mL) or BP (200 μM) was added to 1 mM H_2_O_2_-supplemented DMEM, incubated for 30 min in a CO_2_ incubator. The concentrations of H_2_O_2_ were then assayed. Data are shown as means ± SD (n = 3). ^‡‡‡^*p* < 0.001 vs. H_2_O_2_-supplemented DMEM only. (**c**) A549 cells were treated for 6 h with the reagents described above in a CO_2_ incubator, followed by the assay of intracellular ROS by the DCF fluorescence method. Data are shown as means ± SD (n = 3). **p < 0.01 vs. DMEM only (v), ^#^*p* < 0.05, ^##^*p* < 0.01 vs. PAM only.

We previously reported that PAM induced A549 cell death, and this ability of PAM was similar to that of 1 mM H_2_O_2_^10^. ROS such as H_2_O_2_ or its derived species may play a role in PAM-mediated injury. We used PAM prepared with Sigma Dulbecco’s modified Eagle’s medium (DMEM) #5796 in the present study, unless specifically stated otherwise. Cell injury, detected by lactate dehydrogenase (LDH) activity released in conditioned medium, was induced by the treatment with PAM, and was significantly attenuated by the Fe(II) chelator 2,2′-bipyridyl (BP; Wako Pure Chemicals, Osaka, Japan), as shown in Fig. 1a. BP also attenuated H_2_O_2_-induced cell injury to a similar extent (Fig. 1a right), but did not exhibit the ability to decompose H_2_O_2_ directly (Fig. 1b). Moreover, the PAM treatment induced the accumulation of ROS (Fig. 1c), while BP and catalase significantly suppressed it. On the other hand, FeCl_2_ added extracellularly did not induce the release of LDH or accumulation of ROS, whereas H_2_O_2_-supplemented medium did.

### Elevations in Fe(II)-specific fluorescence by the PAM treatment

**Figure 2:**
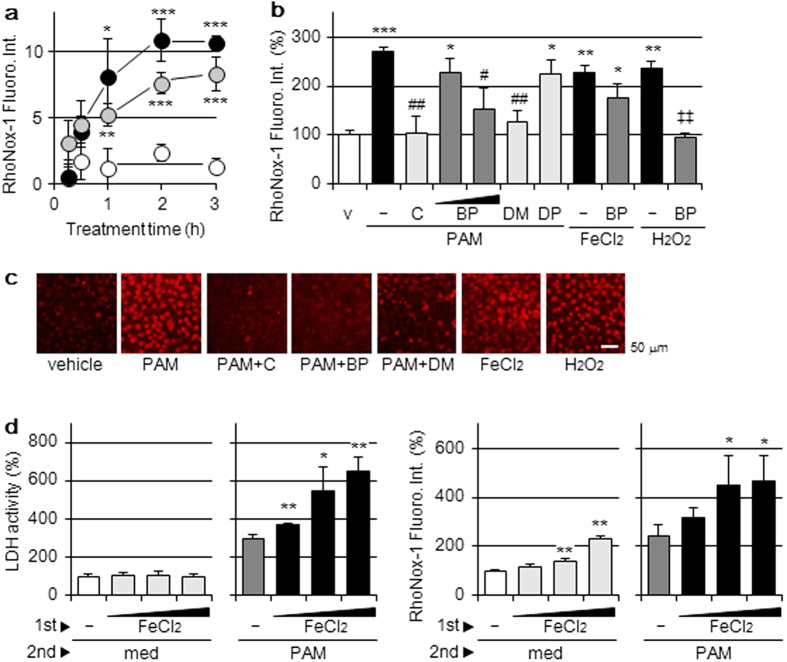
Elevations in Fe(II)-specific fluorescence by the PAM treatment. (**a**) A549 cells were treated with DMEM (open circle), PAM (closed circle), or 100 μM FeCl_2_-supplemented DMEM (gray circle) for the indicated hours in a CO_2_ incubator, followed by the assay of intracellular Fe(II) levels with RhoNox-1. Data are shown as means ± SD (n = 4). *p < 0.05, **p < 0.01, ***p < 0.001 vs. DMEM. (**b**) A549 cells were treated with DMEM (v); PAM in the presence or absence of catalase (50 U/mL), BP (50, 200 μM), DMTU (10 mM), or DPQ (20 μM); FeCl_2_-supplemented DMEM (100 μM) in the presence or absence of BP (200 μM), or H_2_O_2_-supplemented DMEM (1 mM) in the presence or absence of BP (200 μM) for 2 h in a CO_2_ incubator, followed by the assay of intracellular Fe(II) levels. Data are shown as means ± SD (n = 3). *p < 0.05, **p < 0.01, ***p < 0.001 vs. DMEM only (v), ^#^*p* < 0.05, ^##^*p* < 0.01 vs. PAM only, ^‡‡^*p* < 0.01 vs. H_2_O_2_-supplemented DMEM only. (**c**) A549 cells were treated for 2 h with the reagents described above in a CO_2_ incubator, followed by confocal laser fluorescence microscopic observations with RhoNox-1. Scale bars, 50 μm. (**d**) A549 cells were treated with DMEM containing 10%FCS and FeCl_2_ (50, 100, 200 μM) for 16 h (1st step) and then with DMEM (med) or PAM for 6 h in a CO_2_ incubator (2nd step). Assays for LDH activity released into the conditioned medium (left panel) and intracellular Fe(II) levels (right panel) were then conducted. Data are shown as means ± SD (n = 4). *p < 0.05, **p < 0.01, vs. without FeCl_2_.

Intracellular Fe(II) was detected using RhoNox-1, a highly selective fluorescent probe^16^. The intensity of the fluorescence signal induced by the treatment of A549 cells with PAM increased in a time-dependent manner by 2 h, as shown in Fig. 2a. The elevation observed in the intensity of fluorescence by the PAM treatment was significantly suppressed by the addition of catalase or BP to PAM prior to the loading of RhoNox-1 (Fig. 2b). This elevation was also observed by the treatment with H_2_O_2_-supplemented medium and was significantly suppressed by BP (Fig. 2b). The treatment of A549 cells with FeCl_2_ induced time-dependent elevations in the intensity of the fluorescence signal; however, this was not suppressed by the addition of BP. Changes in RhoNox-1 fluorescence by the treatment with PAM and/or other reagents were confirmed by confocal laser fluorescence microscopic observations (Fig. 2c).

The treatment of A549 cells, which had already been treated with various concentrations of FeCl_2_, with PAM induced the release of LDH in a FeCl_2_ concentration-dependent manner, as shown in Fig. 2d left panel, and elevations in the release of LDH were associated with increases in intracellular Fe(II) levels (Fig. 2d right). On the other hand, increases in the release of LDH were not observed when these cells were treated with DMEM; however, the intracellular level of Fe(II) was elevated.

### Contribution of intracellular Fe(II) on cell injury

**Figure 3:**
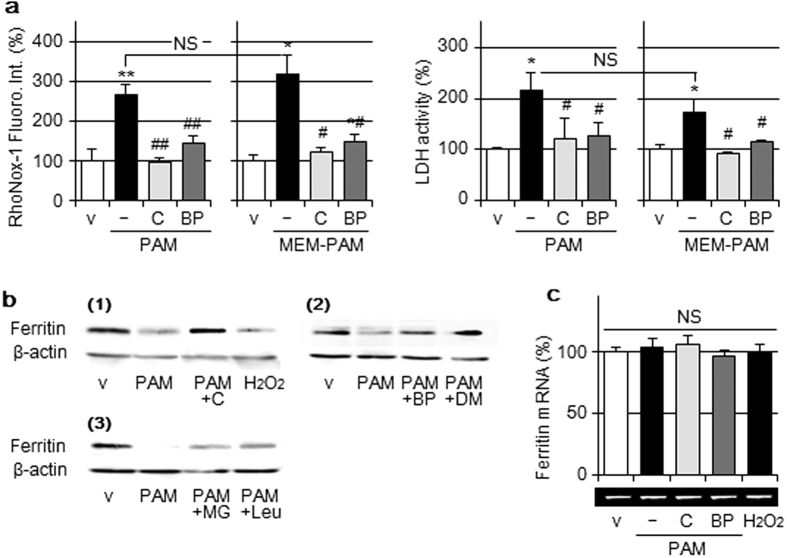
Contribution of intracellular Fe(II) on cell injury. (**a**) A549 cells were treated with DMEM or MEM (v); PAM or MEM-PAM in the presence or absence of catalase (50 U/mL) or BP (200 μM) for 3 h in a CO_2_ incubator, followed by the assay of intracellular Fe(II) levels (left panel) or released LDH activity (right panel). Data are shown as means ± SD (n = 3). *p < 0.05, **p < 0.01 vs. vehicle, ^#^*p* < 0.05, ^##^*p* < 0.01 vs. PAM or MEM-PAM only, NS not significant. (**b**) (1) A549 cells were treated with DMEM (v), PAM in the presence or absence of catalase (50 U/mL), or H_2_O_2_-supplemented DMEM (1 mM) for 3 h in a CO_2_ incubator, followed by Western blotting for the ferritin H chain and β-actin. (2) A549 cells were treated with DMEM (v), PAM in the presence or absence of BP (200 μM) or DMTU (10 mM) for 3 h in a CO_2_ incubator, followed by Western blotting. (3) A549 cells were pretreated with MG132 (10 μM) or leupeptin (10 μM) for 3 h and then treated with DMEM (v), PAM in the presence or absence of MG132 or leupeptin for 3 h in a CO_2_ incubator, followed by Western blotting. (**c**) A549 cells were treated for 3 h with the reagents described above in a CO_2_ incubator, followed by RT-PCR for the ferritin H chain. RT-PCR data were normalized using β-actin levels. Data are shown as means ± SD (n = 3), NS not significant.

The ability of PAM prepared using Sigma minimum essential medium (MEM) #2279 under the same plasma irradiation conditions (MEM-PAM) to elevate intracellular Fe(II) was similar to that of PAM (prepared using Sigma DMEM #5796) (Fig. 3a left). MEM #2279 did not contain iron ions, whereas DMEM #5796 was formulated with 0.1 μg/mL ferric nitrate. MEM-PAM-induced cell injury (LDH-release) was not significantly difference from that induced by PAM (Fig. 3a right). MEM-PAM-induced elevations in intracellular Fe(II) and cell injury were significantly attenuated by the addition of catalase or BP.

Ferritin is an iron storage protein that regulates cellular iron homeostasis^13^,^14^. Ferritin protein levels were reduced by the PAM treatment and this was attenuated by the addition of catalase to PAM, as shown in Fig. 3b-(1), whereas ferritin mRNA levels remained unchanged (Fig. 3c). The treatment of cells with H_2_O_2_-supplemented medium also decreased ferritin protein levels. The addition of BP to PAM attenuated reductions in ferritin protein levels (Fig. 3b-2). Moreover, reductions in ferritin protein levels by the PAM treatment were attenuated by the pretreatment of cells with MG132 (Calbiochem), a proteasome inhibitor or leupeptin (Peptide Institute, Osaka, Japan), a lysosomal proteinase inhibitor (Fig. 3b-3).

### Hydroxyl radical generation by the PAM treatment

**Figure 4:**
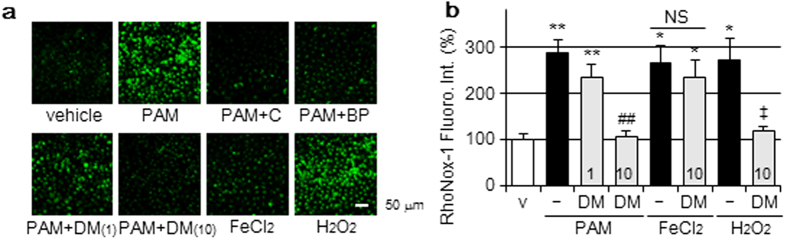
Hydroxyl radical generation by the PAM treatment. (**a**) A549 cells were treated with DMEM (vehicle); PAM in the presence or absence of catalase (50 U/mL), BP (200 μM), or DMTU (1, 10 mM); FeCl_2_-supplemented DMEM (100 μM) or H_2_O_2_-supplemented DMEM (1 mM) for 30 min in a CO_2_ incubator, followed by confocal laser fluorescence microscopic observations with HPF. Scale bars, 50 μm. (**b**) A549 cells were treated with DMEM (v), PAM, FeCl_2_-supplemented DMEM (100 μM), or H_2_O_2_-supplemented DMEM (1 mM) in the presence or absence of DMTU for 2 h in a CO_2_ incubator, followed by the assay of intracellular Fe(II) levels. Numbers in columns show the concentration (mM) of DMTU. Data are shown as means ± SD (n = 3). *p < 0.05, **p < 0.01 vs. DMEM only (v), ^##^*p* < 0.01 vs. PAM only, ^‡^*p* < 0.05 vs. H_2_O_2_-supplemented DMEM only, NS not significant.

Hydroxyl radical (•OH) is generally considered to be the most potent ROS generated from H_2_O_2_ and Fe(II) by the Fenton reaction and reacts rapidly with DNA, resulting in its fragmentation. H_2_O_2_ is the major active component of PAM and freely passes through the plasma membrane to induce cellular injury^17^. Elevations in the intensity of RhoNox-1 fluorescence by the PAM treatment (Fig. 2) prompted us to determine •OH with the specific probe 2-[6-(4′-hydroxy) phenoxy-3H-xanthen-3-on-9-yl] benzoic acid (HPF)^18^. The PAM treatment increased the intensity of intracellular HPF fluorescence and this was suppressed by the addition of catalase or BP, as shown in Fig. 4a. The treatment of A549 cells with H_2_O_2_-supplemented medium also elevated the intensity of HPF fluorescence, whereas the addition of FeCl_2_-supplemented medium did not. The •OH scavenger 1,3-dimethyl-2-thiourea (DMTU, Tokyo Chemical Industry, Tokyo, Japan)^19^ diminished the elevated intensity of HPF fluorescence induced by the PAM treatment (Fig. 4a). The addition of DMTU to PAM also significantly suppressed the release of LDH and accumulation of ROS (Fig. 1), and reduction in ferritin protein level (Fig. 3b-2). Moreover, DMTU significantly inhibited the elevations induced in the intensity of RhoNox-1 fluorescence by PAM or H_2_O_2_-supplemented medium, but not by FeCl_2_-supplemented medium (Fig. 4b).

### Nuclear apoptotic changes by the PAM treatment

**Figure 5:**
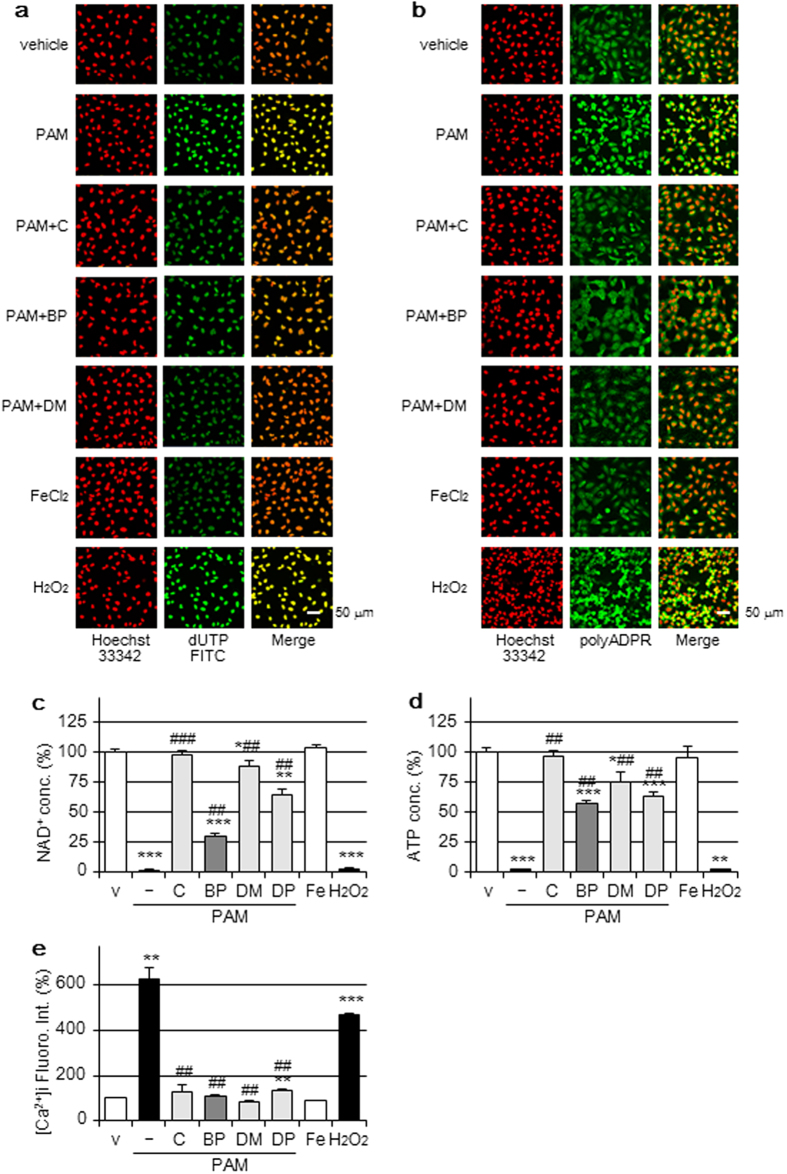
Nuclear injury by the PAM treatment. (**a,b**) A549 cells were treated with DMEM (vehicle); PAM in the presence or absence of catalase (50 U/mL), BP (200 μM), or DMTU (10 mM); FeCl_2_-supplemented DMEM (100 μM) or H_2_O_2_-supplemented DMEM (1 mM) for 2 h in a CO_2_ incubator. The TUNEL assay (**a**) or immunostaining for polyADPR (**b**) was then performed. Scale bars, 50 μm. (**c**,**d**) A549 cells were treated for 2 h with the reagents described above in a CO_2_ incubator, followed by the assay of NAD^+^ (**c**) or ATP (**d**). Data are shown as means ± SD (n = 3). *p < 0.05, **p < 0.01, ***p < 0.001 vs. DMEM only (v), ^##^*p* < 0.01, ^###^*p* < 0.001 vs. PAM only. (**e**) A549 cells were treated for 6 h with the reagents described above in a CO_2_ incubator, followed by the assay of [Ca^2+^]i by the Fluo 4 fluorescence method. Data are shown as means ± SD (n = 3). **p < 0.01, ***p < 0.001 vs. DMEM only (v), ^##^*p* < 0.01 vs. PAM only.

The terminal deoxynucleotidyl transferase-mediated dUTP-biotin nick-end-labeling (TUNEL) assay is a common method for detecting DNA fragmentation resulting from apoptotic signaling cascades. The treatment with PAM increased the number of TUNEL-positive cells, which were also reduced by the addition of catalase, BP, or DMTU as shown in Fig. 5a. H_2_O_2_-supplemented medium also induced TUNEL staining, whereas FeCl_2_-supplemented medium did not.

The accumulation of poly(ADP-ribose) (polyADPR) was subsequently assayed because they are the final products of poly(ADP-ribose) polymerase-1 (PARP-1), which acts as a molecular sensor of DNA-strand breaks and repairs them^20^. The formation of polyADPR was detected in PAM and H_2_O_2_-treated cells and was suppressed by the reagents roughly parallel to TUNEL staining (Fig. 5b).

The over-activation of PARP-1 has been shown to induce the cleavage of NAD^+^ into nicotinamide and ADP-ribose, thereby forming large amounts of polyADPR, which results in the consumption of NAD^+^ and depletion of ATP. As shown in Fig. 5c,d, significant reductions in total cellular NAD^+^ and ATP levels were detected in cells treated with PAM and were significantly attenuated by the addition of catalase, BP, or DMTU. 3,4-dihydro-5-[4-(1-piperidinyl) butoxy]-1(2H)-isoquinolinone (DPQ, EMD Chemicals, San Diego, CA), a PARP-1 inhibitor, also significantly attenuated these reductions. H_2_O_2_-supplemented medium also reduced NAD^+^ and ATP levels, whereas FeCl_2_-supplemented medium did not. The addition of DPQ to PAM significantly suppressed the release of LDH and accumulation of ROS, as shown in Fig. 1, whereas DPQ did not suppress elevations in the intensity of RhoNox-1 fluorescence by PAM (Fig. 2b). The accumulation of ADPR is known to elevate intracellular calcium ion concentrations ([Ca^2+^]i)^21^. PAM-induced elevations in [Ca^2+^]i were significantly suppressed by the addition of catalase, BP, DMTU, or DPQ, as shown in Fig. 5e. On the other hand, FeCl_2_ added extracellularly did not induce elevations in [Ca^2+^]i, whereas H_2_O_2_-supplemented medium did.

### Mitochondrial and endoplasmic reticulum injuries induced by the PAM treatment

**Figure 6:**
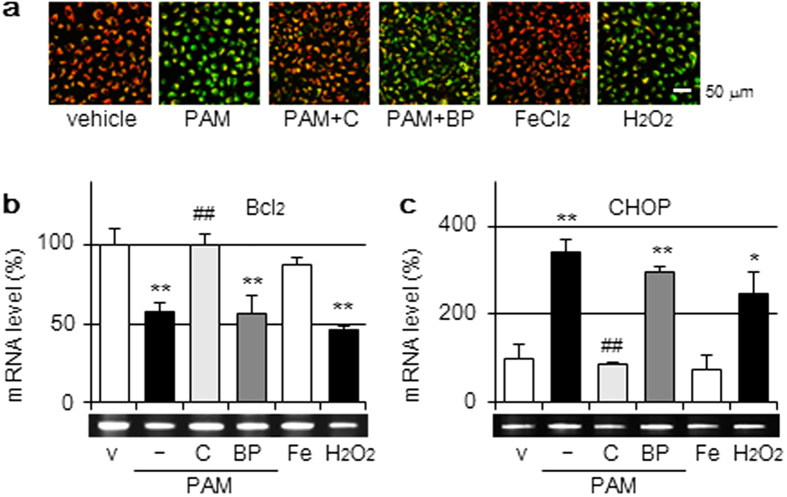
Mitochondrial and endoplasmic reticulum injuries by the PAM treatment. (**a**) A549 cells were treated with DMEM (vehicle); PAM in the presence or absence of catalase (50 U/mL) or BP (200 μM); FeCl_2_-supplemented DMEM (100 μM) or H_2_O_2_-supplemented DMEM (1 mM) for 2 h in a CO_2_ incubator, followed by JC-1 staining. Scale bars, 50 μm. (**b**,**c**) A549 cells were treated for 3 h with the reagents described above in a CO_2_ incubator, followed by RT-PCR for Bcl_2_ (**b**), CHOP (**c**), and β-actin. RT-PCR data were normalized using β-actin levels. Data are shown as means ± SD (n = 3). *p < 0.05, **p < 0.01 vs. DMEM only (v), ^##^*p* < 0.01 vs. PAM only.

We previously demonstrated that the PAM treatment impaired mitochondrial membrane function and changed JC-1 fluorescence^10^. The fluorescence characteristics of JC-1 changed in accordance with mitochondrial membrane potential (Δψm); green fluorescence indicated a decreased Δψm in injured cells, whereas red fluorescence reflected a normal Δψm^22^. As shown in Fig. 6a, decreases in Δψm were observed in PAM or H_2_O_2_-treated A549 cells, but not in FeCl_2_-treated cells. The addition of catalase attenuated the PAM-induced decline in Δψm, whereas that of BP did not.

The expression of anti-apoptotic proteins in mitochondria Bcl_2_ mRNA was significantly decreased by the treatment with PAM and this was abolished with catalase, but not with BP (Fig. 6b). Mitochondrial dysfunction and endoplasmic reticulum (ER) stress interact to disrupt each other and facilitate cellular injury. The expression of C/EBP homologous protein (CHOP), a regulator of apoptosis during ER stress, was induced by PAM. The induction of CHOP was inhibited by catalase, but not by BP (Fig. 6c). H_2_O_2_-supplemented medium changed the expression of these factors, whereas FeCl_2_-supplemented medium did not.

### Selective apoptotic effects of PAM on A549 cells relative to normal cells

**Figure 7:**
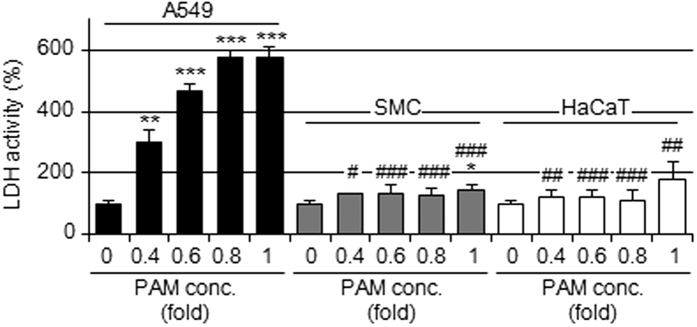
PAM-induced cell injury in normal cells. PAM was diluted and applied to A549 cells (closed column), SMCs (gray column), or HaCaT cells (open column) for 6 h, followed by the assay to assess the viabilities of these cells. Data are shown as means ± SD (n = 3). *p < 0.05, **p < 0.01, ***p < 0.001 vs. DMEM only (0), ^#^*p* < 0.05, ^##^*p* < 0.01, ^###^*p* < 0.001 vs. A549 cells.

We determined whether PAM has selective apoptotic effects on cancer cells. The release of LDH from PAM-treated normal cells such as smooth muscle cells (SMCs) or keratinocytes (HaCaT) was significantly weaker than that from A549 cells, as shown in Fig. 7.

## Discussion

RONS themselves or their derived species are generally considered to be the main bioactive components of PAM. We recently reported that PAM functioned as a donor of reactive species, mainly H_2_O_2_, and induced apoptosis in an A549 cell line^10^. The attenuation of cell death by the addition of iron chelators, beside antioxidants, to PAM was determined and the results obtained prompted us to elucidate the implication of iron ions in cell toxicity induced by PAM.

We detected the significant attenuating effects of the Fe(II) chelator BP on the release of LDH (Fig. 1), •OH production (Fig. 4), nuclear injury, and elevations in [Ca^2+^]i (Fig. 5) induced by PAM. BP is a membrane-permeable Fe(II)-selective chelator and decreases the intensity of fluorescence generated by the reaction of RhoNox-1 with Fe(II) (Fig. 2b,c). The MEM-PAM treatment increased the fluorescence intensity of RhoNox-1 and induced the release of LDH in spite of the absence of iron ions in the MEM formulation (Fig. 3a). These results suggest that the elevations induced in intracellular Fe(II) by the PAM treatment did not depend on the influx of extracellular Fe(II), but on the liberation of intracellularly harbored iron. The result that DMTU almost completely abolished elevations in intracellular Fe(II) by the treatment with PAM or H_2_O_2_-supplemented medium (Fig. 4b) suggested that intracellular •OH led to the liberation of intracellular iron. On the other hand, elevations in intracellular Fe(II) by the FeCl_2_ treatment may depend on the influx of extracellular Fe(II) through the divalent metal transporter-1^23^. The result that DMTU (Fig. 4b) and BP (Fig. 2b) did not abolish elevations in intracellular Fe(II) by the FeCl_2_ treatment were consistent with this finding.

Ferritin is a Fe(III) storage protein that plays crucial roles in cellular and organismal iron homeostasis. However, oxidizing agents containing H_2_O_2_ have been reported to release iron from ferritin shells in the cytosol^24^, which is followed by the degradation of apo-ferritin by the proteasome and/or lysosome systems^13^,^25^. Fe(III) is reduced to Fe(II) following its release from ferritin by a reducing environment^26^,^27^. Reductions in the protein, but not mRNA levels of ferritin by the PAM treatment suggest the degradation of ferritin (Fig. 3b,c). We demonstrated that MG132, a proteasomal proteinase inhibitor, and leupeptin, a lysosomal inhibitor, suppressed the degradation of ferritin (Fig. 3b-3). A number of ferritin degradation pathways that play different roles under various physiological conditions may exist. The result that BP and DMTU also abolished the degradation of ferritin suggested the involvement of •OH rather than H_2_O_2_ in the release of iron. Iron may be released, even from ferritin pores, without the degradation of its structure, and iron-released apo-ferritin is then degraded by proteolysis^28^. The iron released from ferritin may stimulate •OH production and promote the spiral apoptotic process.

Intracellular •OH production has been detected in cells treated with PAM using the specific probe HPF^18^,^29^, as shown in Fig. 4a. This species is produced by the Fenton reaction with intracellular Fe(II) and H_2_O_2_. The result that the treatment of cells with FeCl_2_-suppremented DMEM did not elevate the intensity of HPF fluorescence (Fig. 4a) suggests that the supply of H_2_O_2_ was indispensable for the intracellular generation of •OH. This is supported by the treatment with FeCl_2_-supplemented DMEM not inducing cell injury such as the release of LDH (Figs 1 and 2), DNA fragmentation, activation of PARP1, or elevations in [Ca^2+^]i (Fig. 5); however, it did elevate intracellular Fe(II) (Fig. 2). Intracellularly generated •OH may mainly trigger cell injury in the nuclear fraction because DMTU effectively abolished cell injury, as determined by TUNEL staining, the activation of PARP-1, and the depletion of NAD^+^ and ATP (Fig. 5).

The over-activation of PARP-1 is known to accelerate the consumption of cellular NAD^+^ with the consequent depletion of ATP; however, this enzyme also contributes to DNA repair and the maintenance of genomic stability. The accumulation of polyADPR, products of the PARP-1 reaction, leads to the translocation of apoptosis-inducing factor (AIF) from mitochondria to the nucleus, and has been shown to result in further damage to DNA^30^. The accumulation of AIF surrounding the nucleus has been detected in cells treated with PAM or H_2_O_2_ and was previously shown to be significantly suppressed by the addition of the PARP-1 inhibitor DPQ^10^. The addition of DPQ suppressed not only depletions in NAD^+^ and ATP, but also the release of LDH, accumulation of ROS, and elevations in [Ca^2+^]i (Figs 1 and 5). These results indicate that DNA damage induced by •OH reduces cell viability. On the other hand, the result that DPQ did not affect PAM-induced Fe(II) elevations (Fig. 2b) suggests that Fe(II) functions as an upstream regulator of DNA damage.

Our results showed that the treatment of A549 cells with PAM elevated intracellular Fe(II) and produced •OH; however, the reaction that triggers subsequent signal pathways as the primary event has not yet been identified. The previous elevation induced in intracellular Fe(II) levels by the pretreatment with FeCl_2_ amplified cell injury caused by the supply of H_2_O_2_ in PAM (Fig. 2d). The addition of PAM to A549 cells triggered the production of •OH by the Fenton reaction because these cells contain Fe(II), but not H_2_O_2_ in a steady state. The •OH produced induces the release of Fe(II) from ferritin, which further promotes the reaction. •OH mainly induces DNA injury, and this is followed by the activation of PARP-1 and depletion of NAD^+^ and ATP. The accumulation of ADPR as a product of the activation of PARP-1 may cause the activation of transient receptor potential-melastatin 2 (TRPM2), which triggers the extracellular influx of Ca^2+^ as well as its release from intracellular stores^21^,^31^. On the other hand, mitochondrial and ER injuries such as reductions in Δψm, the down-regulated expression of Bcl_2_, and up-regulated expression of CHOP may be triggered by H_2_O_2_ rather than •OH because BP did not suppressed these changes, whereas catalase completely abolished them (Fig. 6).

Selectivity is one of the most important aspects for the application of plasma to cancer therapy. The sensitivities of SMCs or HaCaT cells to PAM were less than that of A549 cells (Fig. 7). Similar to the findings of previous studies, normal cells are generally more resistant to plasma treatments than cancer cell lines; however, the underlying mechanism has not yet been determined^32^,^33^,^34^,^35^.

**Figure 8:**
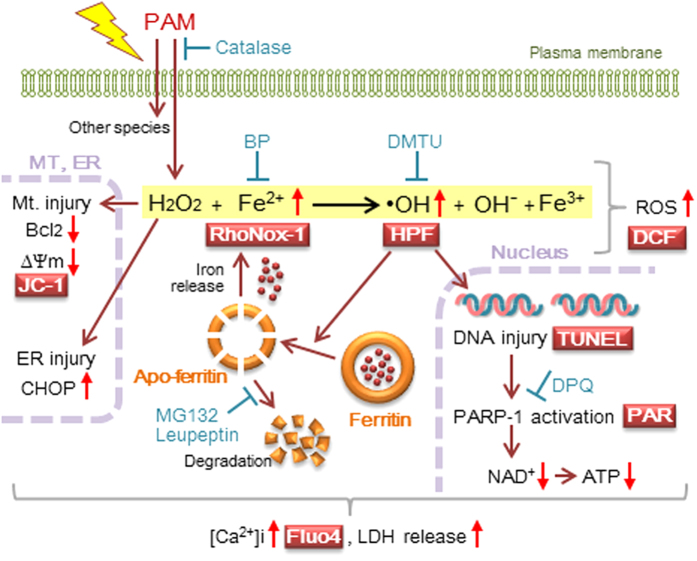
Involvement of iron-triggered •OH in PAM-induced A549 cell injury. The addition of PAM to A549 cells triggers the production of •OH by the Fenton reaction with H_2_O_2_ in PAM and intracellular Fe(II). •OH produced induces the release of Fe(II) from ferritin, which, in turn, further promotes spiral apoptotic reactions. •OH mainly induces DNA fragmentation, followed by the activation of PARP-1 and depletion of NAD^+^ and ATP. On the other hand, mitochondrial and ER injuries such as reductions in Δψm, the down-regulated expression of Bcl_2_, and up-regulated expression of CHOP, may be triggered by H_2_O_2_. These reactions may induce elevations in [Ca^2+^]i and, ultimately, cell death.

Taken together, H_2_O_2_ in PAM and/or its derived •OH in the presence of intracellular iron triggered a spiral apoptotic cascade in the nuclear-mitochondrial network (Fig. 8). The application of PAM to the field of medicine has rapidly expanded recently^6^,^36^,^37^. PAM treatments and plasma irradiation in nude mice bearing cancer cells significantly reduced tumor growth rates and/or improved survival^6^,^38^. The advantages of PAM over direct plasma exposure are that it is possible to prepare in advance for clinical cancer therapy and is administered in a similar manner to medicine. Although further investigations are needed, the results of the present study provide evidence for the anti-tumor effects of PAM and its potential for clinical applications.

## Methods

### Cell culture

A549 cells (human lung adenocarcinoma epithelial cells), SMCs (human aortic smooth muscle cells), and HaCaT cells (human skin keratinocytes) were grown in DMEM supplemented with 10% fetal calf serum (FCS), 100 units/mL penicillin, and 100 μg/mL streptomycin under an atmosphere of 5% CO_2_/95% air at 37 °C.

### Preparation of plasma-activated medium

The experimental setup of the non-thermal atmospheric pressure plasma irradiation system used in this study consisted of a power controller/gas flow regulator, argon (Ar) gas cylinder, and plasma source head (PN-120 TPG, NU Global, Nagoya, Japan) and was the same as the system described previously^6^,^10^. The flow rate of Ar gas was set at 2 standard liters/min (slm). PAM was prepared by exposing plasma to 6 mL of DMEM #5796 (Sigma-Aldrich, St Louis, MO, USA) or MEM #2279 (Sigma-Aldrich), without FCS and antibiotics in 35-mm culture dishes (Nunc #153066). The distance between the plasma source and surface of the media was fixed at L = 3 mm. The duration time for PAM irradiation was 3 min.

### Measurement of cell viability

The LDH-releasing assay was used to determine the effects of PAM on cell viability. Cells were cultured in a 96-well microplate (Nunc #167008) for 24 h in a CO_2_ incubator and then used in experiments. After the treatment of cells with 80 μL of PAM in a 96-well microplate for the indicated hours in a CO_2_ incubator, the activity of LDH released to conditioned medium was assayed using the LDH cytotoxic test (Wako Pure Chemical, Osaka, Japan) according to the manufacturer’s directions.

### Assays to determine H_2_O_2_ concentrations

H_2_O_2_ concentrations in medium were assayed by a colorimetric method using 3-methyl-2-benzothiazolinone hydrazine hydrochloride, N,N-dimethylaniline, and horseradish peroxidase^39^.

### Detection of ROS generation

The intracellular generation of ROS was quantified by the method described in our previous study^40^ with minor modifications. A549 cells in a 96-well microplate (seeded at 2 × 10^4^ cells/well) were cultured for 24 h in a CO_2_ incubator and then used in experiments. After the treatment of A549 cells with PAM (80 μL) for the indicated hours in a CO_2_ incubator, cells were washed once with phosphate-buffered saline (PBS) and then incubated with fresh medium without FCS and containing 10 μM 5-(and-6)-carboxy-2′,7′-dichlorodihydrofluorescein diacetate (Invitrogen, Carlsbad, CA, USA) for 30 min in the CO_2_ incubator. After this incubation, cells were washed once with PBS and the fluorescence intensity of 2′,7′-dichlorodihydrofluorescein (DCF) was assayed (excitation, 485 nm; emission, 520 nm). The intensity of fluorescence was normalized relative to the cellular protein level in each sample.

The intracellular generation of •OH was detected using HPF according to a previously reported method^41^. A549 cells in a 4-well plate (Nunc #176740, seeded at 1 × 10^5^ cells/well) were cultured for 24 h in a CO_2_ incubator and then used in experiments. After the treatment of cells with PAM (250 μL) in the presence of 10 μM HPF (Sekisui Medical, Tokyo, Japan) for 30 min in the CO_2_ incubator, the medium was replaced with 10 μM HPF-supplemented Hank’s balanced salt solution (HBSS) and incubated for 30 min in the CO_2_ incubator. After this incubation, cells were washed once with HBSS and the cells were visualized under an LSM 710 confocal laser fluorescence microscope (Carl Zeiss, Gottingen, Germany).

### Detection of ferrous ions

RhoNox-1, a turn-on fluorescent probe for the selective detection of ferrous iron, Fe(II), was prepared as described previously^16^ and preserved at −80 °C. A549 cells in a 96-well microplate (seeded at 2 × 10^4^ cells/well) were cultured for 24 h in a CO_2_ incubator and then used in experiments. After the treatment of cells with PAM (80 μL) for the indicated hours in a CO_2_ incubator, cells were washed once with PBS and then incubated with 5 μM RhoNox-1 in DMEM #1145 (Sigma-Aldrich) for 1 h in the CO_2_ incubator. Fluorescence intensity was assayed (excitation, 525 nm; emission, 580 nm) after cells were washed once with PBS. The intensity of fluorescence was normalized relative to the cellular protein level in each sample.

A549 cells in a 4-well culture plate (seeded at 1 × 10^5^ cells/well) were cultured for 24 h in a CO_2_ incubator and then used in experiments. Cells were treated with PAM (400 μL), as described above, and RhoNox-1 fluorescence-positive cells were visualized under an LSM 710 confocal laser fluorescence microscope (Carl Zeiss).

### Polymerase chain reaction analysis

A549 cells in 60-mm culture dishes (Nunc #150288, seeded at 5 × 10^5^ cells/dish) were cultured for 24 h in a CO_2_ incubator and then used in experiments. After the treatment of cells with PAM (4 mL) for the indicated hours in a CO_2_ incubator, the cells were washed once with cold PBS and total RNA was then extracted from cells with 1 mL of TRIzol reagent (Invitrogen). The preparation of cDNA and reverse transcriptional-polymerase chain reaction (RT-PCR) were performed using the methods described in our previous study^42^. The primers for RT-PCR were as follows: ferritin H chain, sense 5′-ATC AAC CGC CAG ATC AAC CT-3′; antisense 5′-TGG CTT TCA CCT GCT CAT TC-3′: Bcl_2_, sense 5′-GAT GTC CAG CCA GCT GCA CCT G-3′; antisense 5′-CAC AAA GGC ATC CCA GCC TCC-3′: CHOP, sense 5′-CCT TCC AGT GTG TGG GAC TT-3′; antisense 5′-GTG TGT TTT CCT TTT GCC GT-3′: β-actin, sense 5′-CAA GAG ATH GCC ACG GCT GCT-3′; antisense 5′-TCC TTC TGC ATC CTG TCG GCA-3′. We ascertained that there was a linear correlation between the amounts of PCR products and template cDNA under our PCR conditions. Aliquots of the PCR mixture were separated on a 2% agarose gel and stained with ethidium bromide. A densitometric analysis of the PCR products was performed with Multi Gauge version 3.0 (Fuji Film, Tokyo, Japan).

### Western blotting

A549 cells in 90-mm culture dishes (Nunc #150350, seeded at 2 × 10^6^ cells/dish) were cultured for 24 h in a CO_2_ incubator and then used in experiments. After the treatment of cells with PAM (8 mL) for the indicated hours in a CO_2_ incubator, the cells were washed with cold PBS, scraped, and lysed in 200 μL of lysis buffer (20 mM Tris-HCl, pH 7.4, containing 1 mM EDTA, 1 mM EGTA, 10 mM NaF, 1 mM Na_3_VO_4_, 20 mM β-glycerophosphate, 1 mM phenylmethylsulfonyl fluoride, 1 mM dithiothreitol (DTT), 2 μg/mL leupeptin, and 1% Triton X-100), followed by centrifugation at 17000 × g for 5 min. After centrifugation, the protein concentration of the supernatant was assayed using a Bio-Rad protein assay reagent. Extracts containing 20 μg of protein were boiled with sample buffer (62.5 mM Tris-HCl, pH 6.8, containing 2% sodium dodecylsulfate (SDS), 10% glycerol, 50 mM DTT, and 0.01% bromophenol blue) for 5 min and separated by SDS-PAGE on a 15% (w/v) polyacrylamide gel. After the proteins were transferred electrophoretically onto polyvinylidene difluoride membranes, non-specific binding sites were blocked with PBS containing 1% bovine serum albumin (BSA). The membranes were then incubated with the respective specific primary antibodies (1:1000). A ferritin H chain antibody from Cell Signaling Technology (Danvers, MA, USA) was used. After the membranes had been washed with PBS containing 0.1% Tween 20, the blots were incubated with a biotin-conjugated goat anti-rabbit IgG antibody (1:1000; Zymed Laboratories, South San Francisco, CA, USA), followed by an incubation with ABC reagents (1:5000; Vector Laboratories, Burlingame, CA, USA). Bands were detected using SuperSignal West Pico (Thermo Scientific) and imaged using LAS-3000 UV mini (Fuji Film).

### Detection of apoptosis

The TUNEL assay was performed to detect apoptotic nuclei. A549 cells were seeded on collagen-coated coverslips (12 mm in diameter) placed in a 4-well culture plate (seeded at 1 × 10^5^ cells/well), cultured for 24 h in a CO_2_ incubator, and then used in experiments. After the treatment of cells with PAM (400 μL) in a CO_2_ incubator for the indicated hours, the cells were washed with PBS followed by fixation with 3% paraformaldehyde and permeabilization with 0.1% Triton X-100. The TUNEL assay was then performed using the Mebstain Apoptosis TUNEL kit (Medical & Biological Laboratories, Nagoya, Japan) according to the manufacturer’s instructions. After the labeling of cell nuclei with Hoechst 33342 (1:1000; Dojindo), cells were washed and visualized under the LSM710 confocal laser fluorescence microscope.

The Δψm was detected using the fluorescence dye JC-1 (Enzo Life Sciences, Farmingdale, NY, USA). The change from red to green fluorescence in JC-1 was used to detect reductions in Δψm. A549 cells in a 4-well culture plate (seeded at 1 × 10^5^ cells/well) were cultured for 24 h in a CO_2_ incubator and then used in experiments. After the treatment of cells with PAM (400 μL) for the indicated hours in a CO_2_ incubator, cells were washed once with PBS followed by the addition of 1 μM JC-1 in 10% FCS-added DMEM. After being incubated for 30 min in a CO_2_ incubator, the cells were washed once with PBS and visualized under an LSM 710 confocal laser fluorescence microscope.

### Detection of poly(ADP-ribose) polymerase-1 activity

PARP-1 activity was detected by the immunostaining of polyADPR, a product of this enzymatic reaction. A549 cells were seeded on collagen-coated coverslips (12 mm in diameter) placed in a 4-well culture plate (seeded at 1 × 10^5^ cells/well), cultured for 24 h in a CO_2_ incubator, and then used in experiments. After the treatment of cells with PAM (400 μL) in a CO_2_ incubator for the indicated hours, the cells were washed with PBS followed by fixation with 3% paraformaldehyde, permeabilization with 0.1% Triton X-100, and then blocked with 3% BSA solution. Cells were incubated for 1 h with an anti-polyADPR (10H) mouse IgG monoclonal antibody (1:50; Immuno-Biological Laboratories, Fujioka, Japan) diluted with Can Get Signal Immunostain solution (Toyobo, Ootsu, Japan). After the cells had been washed with PBS, they were incubated for 1 h with Alexa Fluor 488 goat anti-mouse IgG antibody (1:400; Invitrogen) diluted with Can Get Signal Immunostain solution. After the labeling of cell nuclei with Hoechst 33342 (1:1000), cells were washed and visualized under the LSM710 confocal laser fluorescence microscope.

### Determination of NAD^+^ and ATP

A549 cells in a 48-well plate (seeded at 6 × 10^4^ cells/well) were cultured for 24 h in a CO_2_ incubator and then used for the determination of NAD^+^ and ATP. After the treatment of cells with PAM (250 μL) for the indicated hours in a CO_2_ incubator, cells were washed with PBS followed by the assay for cellular NAD^+^ using the EnzyChrom NAD^+^/NADH assay kit (BioAssay System, Hayward, CA, USA) according to the manufacturer’s directions. Changes in intracellular ATP were also determined after the treatment of A549 cells with PAM using the ENLITEN ATP assay system (Promega, Madison, WI, USA) according to the manufacturer’s directions.

### Assay of intracellular calcium

A549 cells (seeded at 2 × 10^4^ cells/well) in a 96-well microplate were cultured for 24 h in a CO_2_ incubator and then used in experiments. After the treatment of cells with PAM (80 μL) for the indicated hours in a CO_2_ incubator, cells were washed once with PBS and the subjected to the assay of [Ca^2+^]i using Calcium Kit-Fluo 4 (Dojindo, Kumamoto, Japan) according to the manufacturer’s directions. The intensity of fluorescence was normalized relative to the cellular protein level in each sample.

### Data analysis

Data are presented as the mean ± SD from at least three experiments. Data were analyzed by the Welch *t*-test. A *p* value of less than 0.05 was considered significant.

## Additional Information

**How to cite this article**: Adachi, T. *et al.* Iron stimulates plasma-activated medium-induced A549 cell injury. *Sci. Rep.* **6**, 20928; doi: 10.1038/srep20928 (2016).
